# A kinase-independent biological activity for insulin growth factor-1 receptor (IGF-1R): Implications for Inhibition of the IGF-1R signal

**DOI:** 10.18632/oncotarget.886

**Published:** 2013-03-23

**Authors:** Filip Janku, Helen J. Huang, Laura S. Angelo, Razelle Kurzrock

**Affiliations:** ^1^ Department of Investigational Cancer Therapeutics (Phase I Clinical Trials Program), The University of Texas MD Anderson Cancer Center, Houston, Texas; ^2^ Moores Cancer Center, The University of California San Diego, La Jolla, California

**Keywords:** IGF-1R, OSI-906, intracellular glucose, autophagy, MCF7 cells

## Abstract

It has been demonstrated that epidermal growth factor receptor (EGFR) can have kinase independent activity. EGFR kinase-independent function maintains intracellular glucose levels via sodium glucose transporter protein 1 (SGLT1) and supports cell survival. It is plausible that this phenomenon can apply to other receptor tyrosine kinases. We found that transfection of insulin-like growth factor receptor (IGF-1R) siRNA into HEK293 (human embryonic kidney) and MCF7 (metastatic breast cancer) cells results in decreased intracellular glucose levels, whereas treatment with the IGF-1R tyrosine kinase inhibitor OSI-906 did not affect intracellular glucose levels. In addition, IGF-1R interacted with SGLT1 in a manner similar to that previously reported with EGFR. The combination of IGF-1R siRNA and OSI-906 resulted in decreased viability of HEK293 and MCF7 cell lines compared to either agent alone. Collectively, these experiments suggest that IGF-1R, has kinase-independent biologic functions and provide a rationale for combining anti-IGF-1R antibodies or siRNA and IGF-1R small molecule inhibitors.

## INTRODUCTION

Insulin-like growth factor-1 receptor (IGF-1R) signaling is an important metabolic pathway in cancer.[1] IGF-1R inhibitors have shown promise in the clinic in several different malignancies, including Ewing's sarcoma, non-small cell lung carcinoma, and adrenocortical carcinoma.[2-5] Both anti-IGF-1R antibodies and small molecules directed against the kinase activity of IGF-1R are being assessed in clinical trials.[4, 6-13] Anti-IGF-1R antibodies down-regulate cell-surface IGF-1R, thereby preventing signaling through IGF-1R. Small molecule kinase inhibitors, such as OSI-906, inhibit the phosphorylation and downstream signaling of IGF-1R.[14] While these molecules target two distinct points in the IGF-1 signaling pathway, the end result is thought to be the same, i.e. down-regulation of signaling through IGF-1R.

A recent publication demonstrated that epidermal growth factor receptor (EGFR) signals through both kinase-dependent and kinase-independent pathways.[15, 16]. For instance down-regulation of EGFR expression with small interfering (si) RNA resulted in a decrease in intracellular glucose levels, and induction of autophagy, as well as an increase in the sub-G1 population in metastatic prostate cancer cells.[16-18]. These observations have important clinical implications as they suggest that mitigating EGFR signaling may require a combination of small molecule (kinase) inhibitors and antibodies (or siRNA). Indeed, synergy between an EGFR kinase inhibitor and an antibody has been demonstrated in preclinical models, and is being tested in the clinic.[19-23] It is plausible that this phenomenon may not be unique to EGFR and can apply to other transmembrane kinase receptors. In our study, we explored whether IGF-1R also demonstrated kinase-independent activity similar to that found with EGFR.

## RESULTS

### IGF-1R siRNA decreases total IGF-1R, p-IGF-1R, and p-Akt

**Figure 1:**
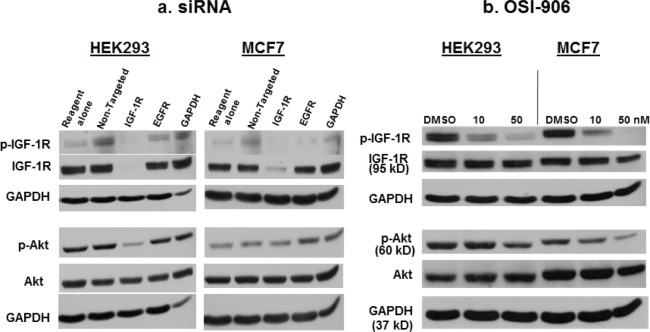
The effect of IGF-1R siRNA or OSI-906 on p-IGF-1R and p-Akt a) IGF-1R siRNA decreases total IGF-1R and p-IGF-1R in HEK293 and MCF7 cells, and p-Akt in HEK293 cells. Cells were transfected with 100 nM siRNA for 48 hours, then lysed for Western blot analysis using appropriate antibodies. EGFR siRNA was used as a control for the specificity of IGF-1R siRNA, and GAPDH siRNA was used as a positive control for transfection. (b) The small molecule kinase inhibitor, OSI-906, inhibits phosphorylation of IGF-1R and Akt in HEK293 and MCF7 cells in a dose-dependent manner. Total IGF-1R levels remain unchanged. Cells were treated for 48 hours, then lysed for Western blot. GAPDH was used as a loading control. Blots are representative of at least three experiments.

HEK293 and MCF7 cells, which both express high levels of IGF-1R, were transfected with siRNA against IGF-1R, and Western blot was performed to confirm the effect of down-regulation of IGF-1R siRNA on p-IGF-1R and p-Akt.[24, 25]. IGF-1R siRNA decreased the expression of total IGF-1R and p-IGF-1R in both cells lines (Figure 1a.), and phosphorylation of the downstream effector Akt was inhibited in HEK293 cells. In MCF7 cells, Akt phosphorylation was not downregulated despite a decrease in total and p-IGF-1R levels, most likely because Akt activation is induced in these cells via both IGF-1R and the insulin receptor (IR).[24]. Transfection with EGFR siRNA served as a control for the specificity of IGF-1R siRNA. GAPDH siRNA served as a positive control for transfection (Figure 1a).

### OSI-906 decreases p-IGF-1R and p-Akt

OSI-906 is a dual kinase inhibitor that inhibits both IGF-1R and IR signaling. MCF7 cells express both IGF-1R and IR (and also IR-A, the fetal isoform of IR).[24] Hence, IGF-1R siRNA was not sufficient to decrease p-Akt in MCF7 cells, but OSI-906 was (Figure 1b). This is consistent with data in the literature suggesting that Akt activation in MCF7 cells involves both IGF-1R and IR. Total IGF-1R remains constant following OSI-906 treatment in both cell lines, while p-Akt and p-IGF-1R are decreased in a dose-dependent manner (Figure 1b).

### IGF-1R siRNA decreases intracellular glucose levels

**Figure 2:**
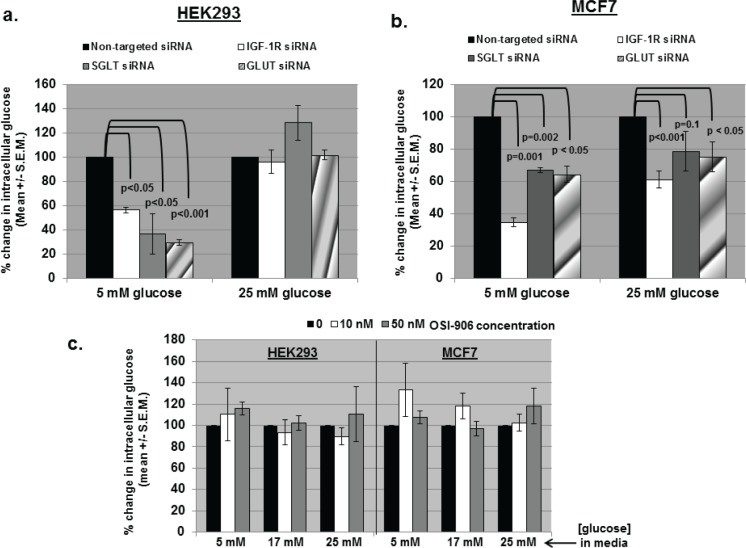
siRNA against IGF-1R, SGLT, and GLUT decrease intracellular glucose levels, but OSI-906 does not (a) IGF-1R, SGLT, and GLUT siRNA significantly decreased intracellular glucose levels in HEK293 cells cultured in low/physiologic (5mM) glucose DMEM, but not in high (25 mM) glucose DMEM. (b) IGF-1R, SGLT, and GLUT siRNA significantly decreased intracellular glucose levels in MCF7 cells cultured in low glucose, and to a lesser extent in high glucose. (c) Intracellular glucose levels were not significantly affected by OSI-906 (10-50 nM) in either cell line in low, medium (17 mM), or high (25 mM) glucose DMEM. All experiments were repeated at least three times and significance determined by student's t test.

Previously, transfection of EGFR siRNA into metastatic prostate cancer cells resulted in decreased intracellular glucose levels, whereas treatment with an EGFR kinase inhibitor did not affect intracellular glucose.[16]. GLUT is a facilitative glucose transporter, and SGLT1 is an active glucose transporter (sodium/glucose cotransporter [SGLT]). These proteins allow glucose to move into and out of a cell.[26, 27] HEK293 and MCF7 cells were transfected with non-targeted, IGF-1R, SGLT1, or GLUT siRNA, and intracellular glucose levels were measured (Figures 2a and 2b). In both cell lines, intracellular glucose decreased significantly after transfection with IGF-1R siRNA. This was demonstrated in HEK293 at low/physiological extracellular glucose levels (5 mM), and was reversible at high (25 mM) glucose levels (Figure 2a). In MCF7 cells, transfection of IGF-1R siRNA decreased intracellular glucose levels at both physiological and high glucose levels (Figure 2b), but was more pronounced at low glucose levels. SGLT1 and GLUT siRNA also decreased intracellular glucose concentrations in both cell lines, and like IGF-1R siRNA, the effect was only seen at low extracellular glucose concentrations in HEK293, and was less pronounced in MCF7 cells.

In contrast to IGF-1R siRNA, OSI-906 did not decrease intracellular glucose levels under any conditions (low 5mM, medium 17 mM, or high 25 mM extracellular glucose) when tested at concentrations up to 1 uM (Figure 2c). Concentrations of 50 nM OSI-906 demonstrated complete (MCF7) or near complete (HEK293) inhibition of IGF-1R kinase activity (Figure 1b), but intracellular glucose levels remained unchanged. These results are analogous to those obtained with EGFR siRNA versus EGFR kinase inhibitors, and indicate that inhibition of the kinase domain of IGF-1R has no effect on the levels of intracellular glucose in either cell line, while down-regulation of total IGF-1R decreases intracellular glucose.[16]

### IGF-1R and SGLT1 interact in HEK293 and MCF7 cells

**Figure 3:**
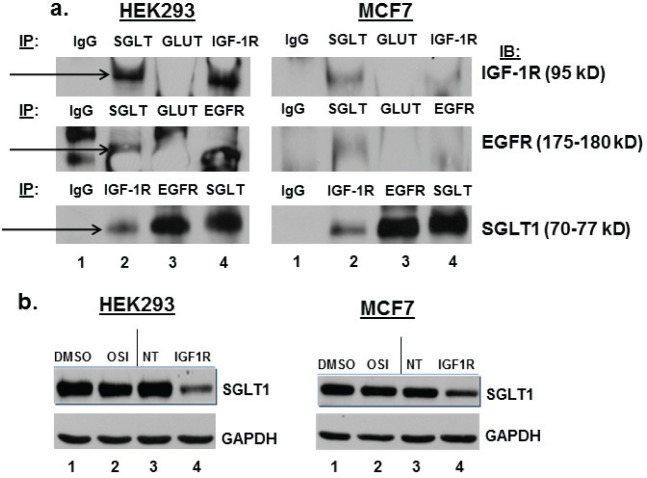
IGF-1R and SGLT1 interact in HEK293 and MCF7 cells, and IGF-1R siRNA transfection results in down-regulation of SGLT1 (a) HEK293 and MCF7 cells were lysed with RIPA buffer, and 500-1000 ug of protein was immuno-precipitated with IgG isotypic control, anti-SGLT1, anti-GLUT, or anti-IGF-1R plus protein A/G beads. Immuno-precipitated extracts were run on an 8% SDS-PAGE gel, immuno-blotted, and probed with anti-IGF-1R, anti-EGFR, or anti-SGLT1. Experiment shown is representative of at least three experiments. Arrows indicate the interaction. (b) HEK293 and MCF7 cells were treated with vehicle (DMSO), 1 uM OSI-906, or transfected with non-targeted siRNA or IGF-1R siRNA for 48 hours. Inhibition of IGF-1R with siRNA also decreases protein expression of SGLT1 (lane 4), whereas OSI-906 has no effect on the expression of SGLT1. GAPDH served as a loading control.

In the paper by Weihua et al., EGFR interacted with SGLT1, but not with GLUT, in PC3MM2 cells.[16] We performed immuno-precipitation followed by Western blot analysis to determine if IGF-1R and SGLT1 also interact endogenously in our cell lines (Figure 3a). When HEK293 or MCF7 cell lysates were immuno-precipitated with anti-SGLT1 and Western blotted with anti-IGF-1R, a strong band appeared (Figure 3a, top, lane 2). This was also true when the antibodies were reversed (Figure 3a, bottom, lane 2). GLUT, however, does not appear to interact with IGF-1R or EGFR (Figure 3a, top and middle panels, lane 3). Cell lysates were immuno-precipitated with anti-EGFR and Western blotted with anti-SGLT1 as a positive control for interaction with SGLT1 (Figure 3a, bottom, lane 3).[16]. In addition to IP/Western blot, interaction of IGF-1R and SGLT-1 was implicated by experiments in which transfection of both cell lines with IGF-1R siRNA also resulted in a decrease in SGLT1 protein expression, whereas non-targeted siRNA did not have this effect (Figure 3b, compare lanes 3 and 4). This is similar to results obtained for EGFR in that down-regulation of total EGFR by siRNA transfection resulted in decreased expression of SGLT1 at the protein level.[16]

### IGF-1R down-regulation does not result in autophagy in HEK293 and MCF7 cells

**Figure 4:**
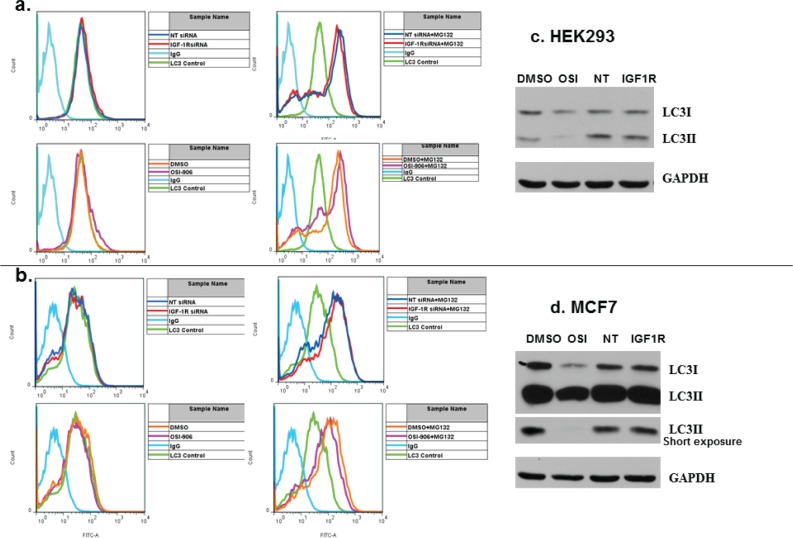
Neither IGF-1R siRNA nor OSI-906 induces the autophagic protein LC3II in HEK293 or MCF7 cells HEK293 and MCF7 cells were transfected with non-targeted or IGF-1R siRNA for 48 hours (top panels, a. and b.) or treated with 1 uM OSI-906 for 48 hours (lower panels, a. and b.). Cells were washed in PBS and immuno-stained with primary anti-LC3B antibody, then stained with FITC-conjugated secondary antibody and subjected to FACS analysis. An IgG antibody was used as an isotypic control (light blue); baseline anti-LC3B staining (light green); non-targeted siRNA (dark blue); IGF-1R siRNA (red); DMSO (orange); OSI-906 (light purple). Cells were treated with 10 uM MG132 overnight as a positive control for autophagy induction (a. and b., bottom panels). LC3B isoforms I and II were also analyzed by Western blot following transfection of HEK293 (c) and MCF7 (d) with siRNA or treatment with 1 uM OSI-906 for 48 hrs. GAPDH expression served as a loading control. NT = non-targeted siRNA.

Since siRNA against EGFR was found to induce autophagic cell death in PC3MM2 cells, we asked whether this was unique to EGFR or if down-regulation of IGF-1R by siRNA could also induce autophagy.[16]. Autophagy typically occurs when the cell is under environmental stress, such as nutrient deprivation, or pathogen infection, and is used to degrade and recycle proteins and organelles as a means of survival, and may result in increased or decreased cell death depending on the circumstances.[17, 18]. Autophagy induction was a possibility since levels of intracellular glucose were decreased by IGF-1R siRNA transfection in a manner similar to that observed for EGFR.[16]. However, IGF-1R siRNA did not induce autophagy in either HEK293 or MCF7 cells. Cells were transfected with non-targeted or IGF-1R siRNA and then immuno-stained for the presence of isoform II of the autophagic protein, microtubule-associated protein 1 light chain 3 (LC3II) (Figure 4a and 4b). Cells were analyzed by flow cytometry. LC3B isoform II typically increases in cells that are undergoing autophagy. Neither IGF-1R siRNA nor OSI-906 induced autophagy in either cell line. MG132, a proteasome inhibitor, was used as a positive inducer of autophagy to show that both cell lines can be induced to increase LC3II expression (Figure 4a and 4b, right panels) even though MCF7 lacks beclin1, a protein important to the autophagic process.[28, 29]. Results were confirmed by Western blot (Figure 4c and 4d). Therefore, unlike EGFR, IGF-1R down-regulation did not result in autophagy in these two cell lines. Interestingly, OSI-906 actually decreased the level of LC3II by Western blot in both cell lines. This result was seen consistently in at least four experiments, indicating that OSI-906 may in fact inhibit autophagy in these cells. Cell cycle analysis was also performed to determine if IGF-1R siRNA increased the sub G1 population (dead/apoptotic cells) as was found for EGFR. Results showed that IGF-1R siRNA did not increase the sub G1 population in either cell line (Supplementary Figure 1).

### IGF-1R siRNA decreases the viability of HEK293 and MCF7 cells alone and in combination with OSI-906

**Figure 5:**
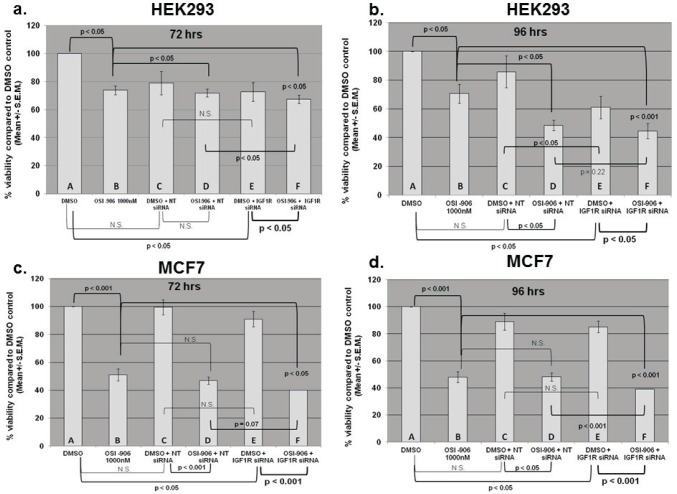
Viability assay results for HEK293 (low glucose) and MCF7 (high glucose) cells transfected with non-targeted or IGF-1R siRNA alone, treated with OSI-906 alone, or various combinations as indicated Cells were transfected with either non-targeted or IGF-1R siRNA (100 nM) for 24 hours, then treated with OSI-906 (1 uM) for an additional 48 or 72 hours, giving final incubation times of 72 or 96 hours. (a) HEK293 - 72 hours; (b) HEK293 - 96 hours; (c) MCF7 - 72 hours; (d) MCF7 - 96 hours. Capital letters were added to the bar graphs for ease of comparison. A= DMSO, B= 1 uM OSI-906 alone, C= DMSO + non-targeted siRNA, D= OSI-906 + NT siRNA, E= DMSO + IGF-1R siRNA, F= OSI-906 + IGF-1R siRNA. Statistical differences were determined by Student's t test. P values <0.05 were considered significant.

We next determined whether IGF-1R siRNA and OSI-906 affected the viability of the cells either alone or in combination. Cells treated with 0.1% DMSO were assigned 100% viability and were used as a negative (vehicle) control. Cells treated with media alone were virtually identical to DMSO only treated cultures. Prior to assessing the effect on viability, the IC50 of OSI-906 on both cell lines was determined (approximately 3 uM for HEK293 at 96 hours and 500-750 nM for MCF7 at 96 hours and 1 uM for MCF7 at 72 hours). Hence all viability and apoptosis experiments were carried out at 1 uM OSI-906 at 72 or 96 hours. A cell viability assay was used to measure the number of viable cells present based on quantification of ATP, which signals the presence of metabolically active cells. The amount of ATP is directly proportional to the number of cells present in culture (see Materials and Methods). Cells were either treated with DMSO as a control (100% viability) or DMSO plus non-targeted siRNA, DMSO plus IGF-1R siRNA, OSI-906 alone, OSI-906 plus non-targeted siRNA, or OSI-906 plus IGF-1R siRNA. Treatment with 1 uM OSI-906 significantly decreased the viability of HEK293 and MCF7 cells by approximately 26% (p<0.05) and 50% (p<0.001) respectively at 72 hours and by 30% (p<0.05) and 52% (p<0.001) respectively at 96 hours (Figure 5, compare bars A and B). Hence, OSI-906 was more effective at decreasing the viability of MCF7 cells than the viability of HEK293 cells at 1 uM. These data correlate with the previously calculated IC50 for each cell line. IGF-1R siRNA alone decreased the viability of HEK293 and MCF7 cells by 28% (p<0.05) and 10% (p<0.05) respectively at 72 hours and 40% (p<0.05) and 15% (p<0.05) at 96 hours (Figure 5, compare bars A and E). Hence, down-regulation of total IGF-1R by siRNA transfection resulted in a statistically significant decrease in the viability of HEK293 cells and MCF7 cells at both time points. Cells were also transfected with non-targeted siRNA as a negative control. There was a non-specific decrease in the viability of both cell lines following transfection of non-targeted siRNA, but this was not statistically significant (Figure 5, compare bars A and C).

The inhibitory effect of OSI-906 alone compared with IGF-1R siRNA alone varied with the cell line and the time of culture. In HEK293 cells, OSI-906 and IGF-1R siRNA did not differ in their ability to decrease viability at 72 hours, but IGF-1R siRNA was more effective than OSI-906 if cultured for 96 hours (Figure 5a and 5b, compare bars B and E). In MCF7 cells, OSI-906 was much more effective (p<0.001, Figure 5c and 5d, compare A and B) than IGF-1R siRNA (p<0.05, Figure 5c and 5d, compare A and E) at decreasing viability at 72 and 96 hours (compare bars B and E), with an enhanced decrease at 96 hours (Figure 5c and 5d)

If IGF-1R has both kinase-dependent and kinase-independent effects, and if these effects are pertinent to cell viability, then the combination of IGF-1R siRNA plus OSI-906 would be expected to decrease the viability of both cell lines more than either agent alone. Figure 5 shows the effect on the viability of HEK293 and MCF7 cells treated with the combination of OSI-906 plus IGF-1R siRNA. Comparison of OSI-906 alone to treatment with OSI-906 plus IGF-1R siRNA (Figure 5, bar B compared to bar F) shows a significant enhancement of the effects of OSI-906 in both cell lines after transfection with IGF-1R siRNA at 72 hours and even more so at 96 hours (HEK293 p<0.05 at 72 hours, p<0.001 at 96 hours; MCF7 p<0.05 at 72 hours, p<0.001 at 96 hours). Alternatively, comparison of DMSO plus IGF-1R siRNA (essentially IGF-1R siRNA alone) to OSI-906 plus IGF-1R siRNA (Figure 5, compare bar E to bar F) also shows a significant decrease in viability of both cell lines at 72 hours (HEK293 p<0.05, MCF7 p<0.001) and 96 hours (HEK293 p<0.05, MCF7 p<0.001). Comparing OSI-906 plus non-targeted siRNA to OSI-906 plus IGF-1R siRNA (Figure 5 compare bar D and F) shows that IGF-1R siRNA is significantly better than non-targeted siRNA when combined with OSI-906 in HEK293 cells at 72 hours (p<0.05) and MCF7 at 96 hours (p<0.001), and shows a trend toward significance in MCF7 cells at 72 hours (p=0.07). A significant difference between the two siRNAs was not seen in HEK293 cells at 96 hours perhaps because the S.E.M. was larger for this time point. Therefore, these results suggest that the combination of the kinase inhibitor OSI-906 and siRNA against IGF-1R have significantly greater suppressive effects on HEK293 and MCF7 viability than either agent alone, though these effects are more pronounced at certain time points.

### OSI-906 and IGF-1R siRNA do not induce apoptosis or increase the subG1 population in HEK293 or MCF7 cells

EGFR siRNA increased the subG_1_ population in transfected metastatic prostate cancer cells [14]. Hence, we treated our cell lines with OSI-906 alone, IGF-1R siRNA alone, or the combination and performed cell cycle analysis. As stated above, there was no significant increase in the subG1 population in either cell line after treatment with OSI-906 or IGF-1R siRNA (Supplementary Figure 1). FITC-Annexin V staining was also performed to determine if our cell lines were undergoing apoptosis after treatment with OSI-906 alone or in combination with IGF-1R siRNA. Baseline levels of apoptosis were slightly higher in MCF7 cells (22%). Treatment of MCF7 with OSI-906 induced only a slight increase in apoptosis (4%) over baseline, and the combination of OSI-906 plus IGF-1R siRNA resulted in a 7% increase in apoptosis compared to OSI-906 alone (not significant by Student's t test, Supplementary Figure 2). Baseline levels of apoptosis were lower in HEK293 cells (10%) and increased to approximately 18% after treatment with OSI-906 alone and increased to only 20% with the combination (not statistically significant by t test, Supplementary Figure 3). Neither OSI-906, IGF-1R siRNA, nor the combination significantly induced apoptosis in MCF7 and HEK293 cells. Together these data indicate that the combination of OSI-906 and IGF-1R siRNA inhibits proliferative pathways, but does not induce apoptotic cell death in the cell lines studied.

## DISCUSSION

Receptor kinases can signal through more than one pathway. For example, CRAF was found to have MEK-independent role, which is critical for mitosis and tumor progression.[15]. In addition, Weihua et al. [15] demonstrated that siRNA directed against EGFR resulted not only in the down-regulation of total EGFR, but also in a decrease in intracellular glucose levels, the induction of autophagic cell death, and an increase in the subG_1_ population. These results were not observed when EGFR kinase activity was inhibited, consistent with the presence of a kinase-independent EGFR signal. Additional preclinical data examining EGFR includes animal models showing that the combination of anti-EGFR antibodies plus EGFR tyrosine kinase inhibitors, such as erlotinib, results in synergistic tumor regression.[19-21]. We therefore asked whether a kinase-independent signal could be observed with other clinically relevant growth factor receptors, such as IGF-1R. Indeed, recent literature suggests that IGF-1R translocates to the nucleus where it is thought to be involved in the transcriptional enhancement of specific target genes, while the kinase domain of IGF-1R is responsible for interaction with other kinases and the activation of intracellular signaling pathways that result in increased proliferation.[30-36]

Our data demonstrate that siRNA directed against total IGF-1R decreases intracellular glucose levels in both HEK293 (human embryonic kidney) and MCF7 (metastatic breast cancer) cell lines, particularly at physiological levels of glucose (5 mM). Higher levels of extracellular glucose can partially attenuate this effect (Figure 2a and b). OSI-906, a small molecule inhibitor of the IGF-1R kinase, did not alter intracellular glucose levels. In addition, IGF-1R was found to interact with the sodium glucose transporter protein SGLT1 in a manner similar to that seen with EGFR. Hence, IGF-1R (like EGFR) may play a role in maintaining intracellular glucose levels in tumor cells in a kinase-independent manner.

In contrast to EGFR, blocking total IGF-1R expression by siRNA transfection did not increase the sub-G_1_ (dead) cell population, nor did it induce autophagy or apoptosis in either cell line following inhibition of IGF-1R expression; however, decreased intracellular glucose levels are not always a prerequisite for autophagy.[37] Even so, the combination of OSI-906 plus IGF-1R siRNA resulted in a greater decrease in cell viability in both cell lines than with either molecule alone (Figure 5). This may have clinical implications in that it suggests that giving an IGF-1R antibody or siRNA together with OSI-906 may be more effective than either agent alone. Of interest, Zeng et al. demonstrated increase in autophagy in breast cancer cell line models (MCF-7, LCC6) sequentially exposed to doxorubicin and IGF-1R/IR tyrosine kinase inhibitor; however, autophagy in these experiments could have been activated by doxorubicin rather than IGF1-R inhibition.[38]

The case for using a monoclonal antibody and a kinase inhibitor together is being studied in the clinic.[22, 23]. We recently demonstrated that 11 of 34 patients (32.4%) achieved stable disease for at least six months and/or partial/complete response with the use of the EGFR kinase inhibitor erlotinib combined with the EGFR antibody cetuximab.[39]. Others have shown that combining the HER2/neu kinase inhibitor lapatinib with a HER2/neu antibody trastuzumab prolonged progression-free survival in patients with HER2/neu positive breast cancer.[40, 41]. Our current results suggest that a similar strategy may be exploitable with IGF-1R inhibition.

There are limitations to this study. For instance, OSI-906 inhibits insulin receptor kinase as well as IGF-1R kinase. It is therefore possible that the more pronounced decrease in cell viability observed when it was combined with siRNA against IGF-1R could be due to the effects on insulin receptor itself. Still, these results suggest that such a combination in the clinic may be worth examining.

In summary, we show that IGF-1R, like EGFR, demonstrates a kinase-independent function. The kinase-independent activity is related to the interaction of IGF-1R with the sodium glucose transporter SGLT1; indeed, down-regulation of IGF-1R suppresses SGLT1, thereby attenuating glucose levels. Unlike EGFR, down-regulation of IGF-1R by siRNA does not result in increased autophagy. However, cell viability is decreased and, furthermore, combining the kinase inhibitor OSI-906 with siRNA against IGF-1R amplifies the suppression of viability, albeit without a significant pro-apoptotic effect. These observations suggest that combining an IGF-1R siRNA (or antibody) with OSI-906 is warranted in the clinical setting. Finally, experiments are underway to determine if other tyrosine kinase receptors also have kinase-independent functions.

## MATERIALS AND METHODS

### Cell lines

HEK293 (human embryonic kidney) and MCF7 (breast adenocarcinoma) cell lines were obtained from American Type Culture Collection (ATCC, Manassas, VA), and were authenticated by short tandem repeat analyses. Both cell lines were maintained in Eagle's Minimal Essential medium (EMEM) (ATCC) plus 10% fetal calf serum (FCS) and maintained in a 37°C incubator with 5% CO_2_. For cultures grown in low (5mM) or high (25 mM) glucose, Hyclone low or high glucose DMEM were used, respectively (LabSource, Romeoville, IL).

### Antibodies and reagents

Primary antibodies used for Western blot included rabbit polyclonal or murine monoclonal antibody against IGF-1R, phosphorylated (p)-IGF-1R (Tyr 1135/1136), Light Chain 3B (LC3B), EGFR, Akt, and p-Akt (ser 473) (Cell Signaling, Danvers, MA), glucose transporter (GLUT1), SGLT1 (Abcam, Cambridge, MA and Millipore, Billerica, MA), and GAPDH (Santa Cruz Biotechnology, Santa Cruz, CA). Secondary antibodies for Western blot included anti-rabbit (Cell Signaling) and anti-mouse (BioRad Laboratories, Hercules, CA) conjugated to horse radish peroxidise (HRP). OSI-906 was purchased from ChemieTek (Indianapolis, IN). Glucose stock (225 mM) was prepared (Invitrogen, Carlsbad, CA) and diluted with culture media.

### siRNA transfection

siRNA transfection was performed to determine the effect of down-regulating IGF-1R and other molecules on cell signaling, survival, autophagy, apoptosis, and intracellular glucose levels. HEK293 (2.5x10^5^) or MCF7 (5x10^5^) cells were seeded per well of a 6 well plate and transfected with DharmaFECT 1 siRNA lipid transfection reagent alone (Dharmacon, Pittsburgh, PA), non-targeted siRNA (siGENOME non-targeting siRNA #1), IGF-1R, EGFR, SGLT, GLUT, or GAPDH siRNA (control) for 48 hours. Following transfection, cell lysates were harvested for Western blot analysis, prepared for an intracellular glucose assay, analyzed by fluorescence activated cell sorter (FACS) analysis, or harvested for viability assay.

### Intracellular glucose assay

Intracellular glucose levels were measured following transfection of cells with IGF-1R siRNA or treatment with OSI-906 to determine the effects of IGF-1R down-regulation or inhibition of IGF-1R tyrosine kinase activity on intracellular glucose levels. 1x10^4^ cells per mL were lysed in Western blot solubilization buffer and mixed with assay reagent (1.5 mM NAD, 1.0 mM ATP, 1.0 unit/ml of hexokinase, and 1.0 unit/ml of glucose-6-phosphate dehydrogenase) according to the manufacturer's instructions (Glucose [HK] assay kit, Sigma, St. Louis, MO). When glucose is phosphorylated by ATP, an equimolar amount of NAD is reduced to NADH. The consequent increase in absorbance at 340 nm of the lysate is directly proportional to glucose concentration.

### Immuno-precipitation and Western blotting

Immuno-precipitation followed by Western blot was performed to determine if there was a physical interaction between IGF-1R and glucose transporter proteins. For Western blots, cells were washed with phosphate buffered saline (PBS) and lysed in 1x RIPA Lysis Buffer (Millipore) plus protease inhibitors. A modified Lowry protein assay was performed (Pierce Biotechnology, Rockford, IL), and equal amounts of protein were loaded onto 7.5-10% SDS-PAGE gels, and then transferred to nitrocellulose membranes by electro-blotting. Blots were blocked in 5.0% non-fat milk, incubated with primary antibody (22°C for 2 hours), washed, and then incubated with an appropriate secondary antibody conjugated to HRP and detected by enhanced chemiluminescence (Amersham Pharmacia Biotech, Piscataway, NJ). For immuno-precipitation experiments, cells were lysed in RIPA buffer (Pierce Biotechnology). 500-1000 ug per lane of lysate was pre-cleared with an isotypic control (Santa Cruz Biotechnology) (4°C for two hours), then incubated with specific antibody plus protein A/G beads (2-4 hours at 4°C), centrifuged, washed 4X with RIPA buffer, resuspended in 2X sample buffer (BioRad), and boiled before loading onto SDS-PAGE gels.

### Analysis of autophagy by LC3B staining and flow cytometry

To determine if cells were undergoing autophagy, levels of the autophagosomal protein LC3B isoform II were measured by staining the cells with an anti-LC3B antibody, and then analyzing by flow cytometry. HEK293 (2.5x10^5^) and MCF7 (5x10^5^) cells were transfected with the appropriate siRNA or treated with DMSO (vehicle) or OSI-906 for 48 hrs, washed in PBS, and fixed by adding 2-4% formaldehyde solution for 10 minutes at 37°C. Cells were then permeabilized by adding ice-cold 100% methanol to pre-chilled cells to a final concentration of 90% and incubated on ice for 30 minutes. 0.5-1x10^6^ cells per tube were washed twice in 0.5% bovine serum albumin (BSA) in PBS. Cells were blocked in 0.5% BSA for 10 minutes at room temperature (RT). Primary antibody (anti-LC3B) was added at a 1:500 dilution and incubated for one hour at RT, then washed with 0.5% BSA. FITC-conjugated secondary antibody (1:1,000) was added and cells were incubated for 30 minutes at RT, washed, and resuspended in 0.5 mL PBS and analyzed by fluorescence activated cell sorter (FACS) analysis (Becton Dickinson, Bedford, MA).

### Viability assay

Viability of the cells was assessed after treating the cells with OSI-906, IGF-1R siRNA, or the combination. 500 HEK293 or 1x10^3^ MCF7 cells were plated per well of an opaque-walled 96 well plate. The CellTiter-Glo® Luminescent Cell Viability Assay (Promega, Madison, WI) was used to measure the number of viable cells present based on quantification of ATP, which signals the presence of metabolically active cells. The amount of ATP is directly proportional to the number of cells present in culture. The assay was performed at RT as described in the manufacturer's instructions. Background luminescence from wells containing media alone was subtracted from experimental wells. 100 uL of CellTiter-Glo ® Reagent was added per well containing 100 uL of culture medium. Contents were mixed on an orbital shaker for 2 minutes to induce cell lysis, and the plate was incubated at RT for 10 minutes to stabilize the luminescent signal. Plates were read on a BioTek Synergy 4 plate reader (Winooski, VT).

### Annexin V apoptosis assay

Following transfection with IGF-1R siRNA or treatment with OSI-906 or the combination, apoptosis was measured by staining cell-surface phosphatidyl serine with FITC-conjugated Annexin V. 2.5x10^5^ HEK293 and 5x10^5^ MCF7 cells were seeded per well of a six well plate and treated for 72 hours, harvested, and stained with FITC-Annexin V and propidium iodide according to the manufacturer's instructions (BD Pharmingen). Cells were analyzed by fluorescence activated cell sorter (FACS) analysis (Becton Dickinson).

### Cell cycle analysis

Cell cycle analysis was performed to determine if there was an increase in the sub G_1_ (dead cell) population following transfection with IGF-1R siRNA or treatment with OSI-906. Cells were fixed in 100% ethanol and stained with propidium iodide, according to the manufacturer's instructions (BD Pharmingen), and then analyzed by FACS analysis.

### Statistical analysis

Student's t-test was used to assess the association between continuous variables. All tests were two-sided, and P values less than 0.05 were considered statistically significant. If not otherwise indicated, error bars in all experiments represent standard deviation error (SE). All statistical analyses were carried out using SPSS 19 computer software (SPSS Chicago, IL).

## Supplementary Figures


