# Supplementary material for: A kinase-independent biological activity for insulin growth factor-1 receptor (IGF-1R): Implications for Inhibition of the IGF-1R signal
Source: Oncotarget. 2013 Mar 23;4(3):463–73. doi: 10.18632/oncotarget.886 (PMC3717308; doi:10.18632/oncotarget.886)

A Kinase-Independent Biological Activity for Insulin Growth Factor-1 Receptor (IGF-1R): Implications for Inhibition of the IGF-1R Signal – Janku et al

Supplementary Figure 1: IGF-1R siRNA or OSI906 did not increase the sub G1 population in HEK293 or MCF7 cells

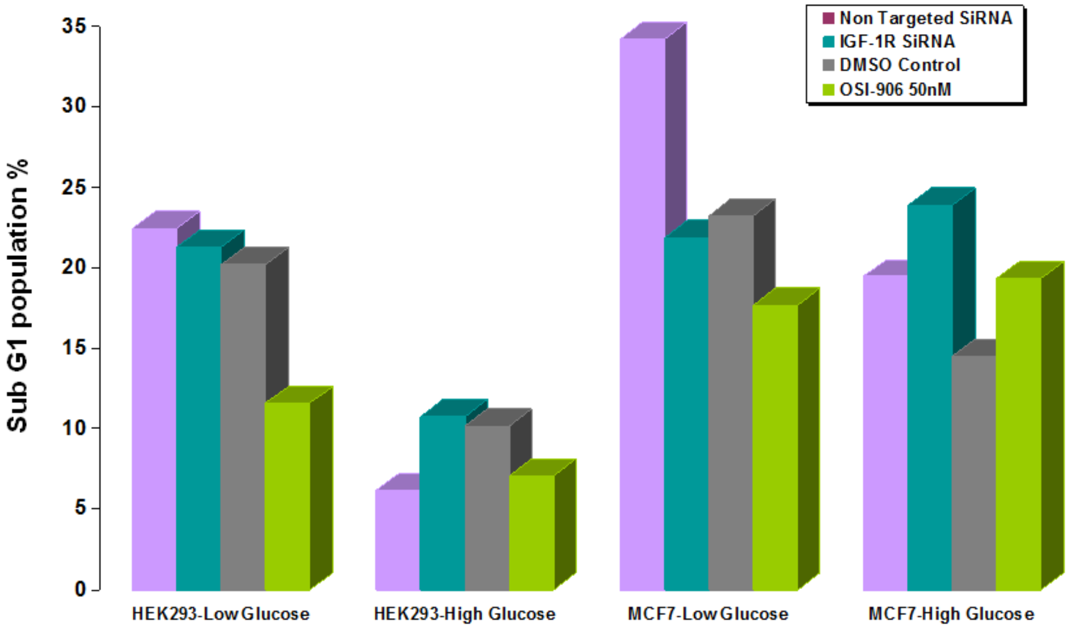

Supplementary Figure 2: Treatment of MCF7 with OSI-906 did not increase apoptosis compared to baseline and the combination of OSI-906 plus IGF-1R siRNA did not increase apoptosis compared to OSI-906 alone.

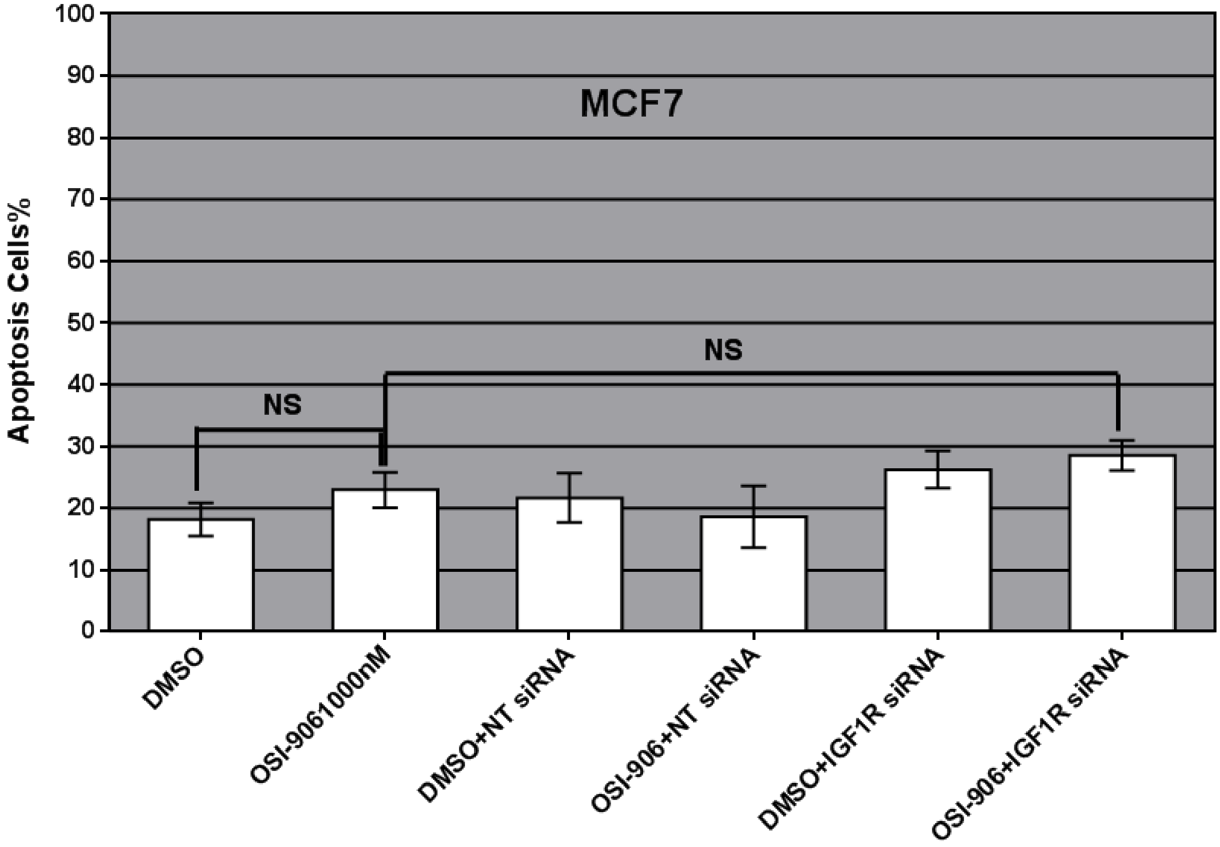

Supplementary Figure 3: Treatment of HEK293 with OSI-906 did not increase apoptosis compared to baseline and the combination of OSI-906 plus IGF-1R siRNA did not increase apoptosis compared to OSI-906 alone.

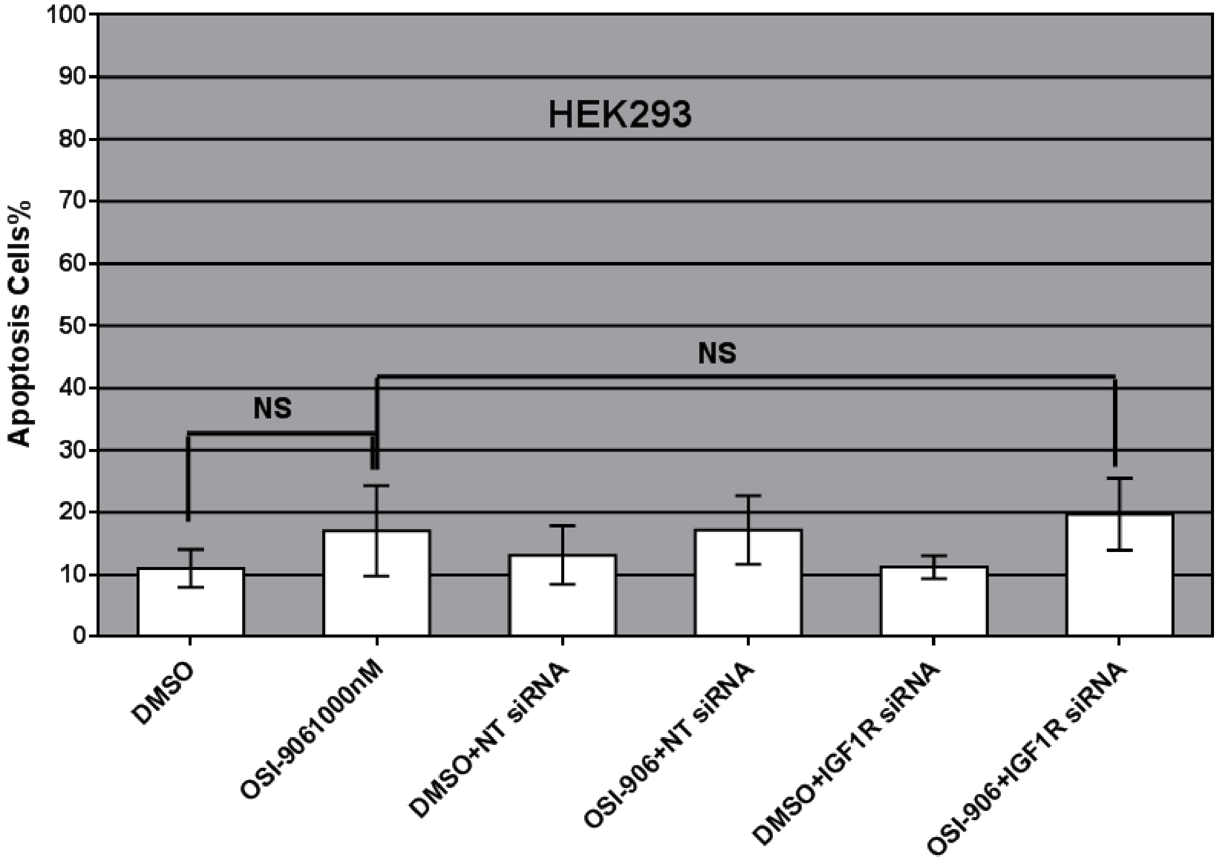

Supplement: Supplementary file 1 [file oncotarget-04-463-s001.pdf]
